# Segmentation of finger tendon and synovial sheath in ultrasound image using deep convolutional neural network

**DOI:** 10.1186/s12938-020-00768-1

**Published:** 2020-04-22

**Authors:** Chan-Pang Kuok, Tai-Hua Yang, Bo-Siang Tsai, I-Ming Jou, Ming-Huwi Horng, Fong-Chin Su, Yung-Nien Sun

**Affiliations:** 1 Department of Computer Science and Information Engineering, 1 University Road, Tainan, 701 Taiwan; 2 Department of Biomedical Engineering, National Cheng Kung University, 1 University Road, Tainan, 701 Taiwan; 3 Department of Orthopedics, E-Da Hospital, 1 Yida Road, Jiaosu Village, Yanchao District, Kaohsiung City, 82445 Taiwan; 4 Department of Computer Science and Information Engineering, National Pingtung University, 4-18 Minsheng Road, Pingtung City, Pingtung County 90003 Taiwan; 5 MOST AI Biomedical Research Center, 1 University Road, Tainan, 701 Taiwan; 6 Department of Orthopaedic Surgery, National Cheng Kung University Hospital, College of Medicine, National Cheng Kung University, 1 University Road, Tainan, Taiwan; 7 Medical Device Innovation Center, National Cheng Kung University, 1 University Road, Tainan, 701 Taiwan

**Keywords:** Convolutional neural network, Segmentation, Synovial sheath, Tendon, Trigger finger, Ultrasound images

## Abstract

**Background:**

Trigger finger is a common hand disease, which is caused by a mismatch in diameter between the tendon and the pulley. Ultrasound images are typically used to diagnose this disease, which are also used to guide surgical treatment. However, background noise and unclear tissue boundaries in the images increase the difficulty of the process. To overcome these problems, a computer-aided tool for the identification of finger tissue is needed.

**Results:**

Two datasets were used for evaluation: one comprised different cases of individual images and another consisting of eight groups of continuous images. Regarding result similarity and contour smoothness, our proposed deeply supervised dilated fully convolutional DenseNet (D2FC-DN) is better than ATASM (the state-of-art segmentation method) and representative CNN methods. As a practical application, our proposed method can be used to build a tendon and synovial sheath model that can be used in a training system for ultrasound-guided trigger finger surgery.

**Conclusion:**

We proposed a D2FC-DN for finger tendon and synovial sheath segmentation in ultrasound images. The segmentation results were remarkably accurate for two datasets. It can be applied to assist the diagnosis of trigger finger by highlighting the tissues and generate models for surgical training systems in the future.

**Methods:**

We propose a novel finger tendon segmentation method for use with ultrasound images that can also be used for synovial sheath segmentation that yields a more complete description for analysis. In this study, a hybrid of effective convolutional neural network techniques are applied, resulting in a deeply supervised dilated fully convolutional DenseNet (D2FC-DN), which displayed excellent segmentation performance on the tendon and synovial sheath.

## Background

Trigger finger is a common hand condition that causes pain, popping, locking and loss of movement of the affected finger [1]. Stretching of fingers, in which the flexor tendon glides through the tendon sheath and the pulley catches the passing tendon close against the bones of the finger [2]. Trigger finger is generally caused by a size mismatch between the flexor tendon and the pulley [3]; e.g., there is swelling in the A1 pulley of the flexor tendon sheath which causes the pulley to be stuck [4]. This situation causes painful triggering or locking of fingers.

**Fig. 1:**
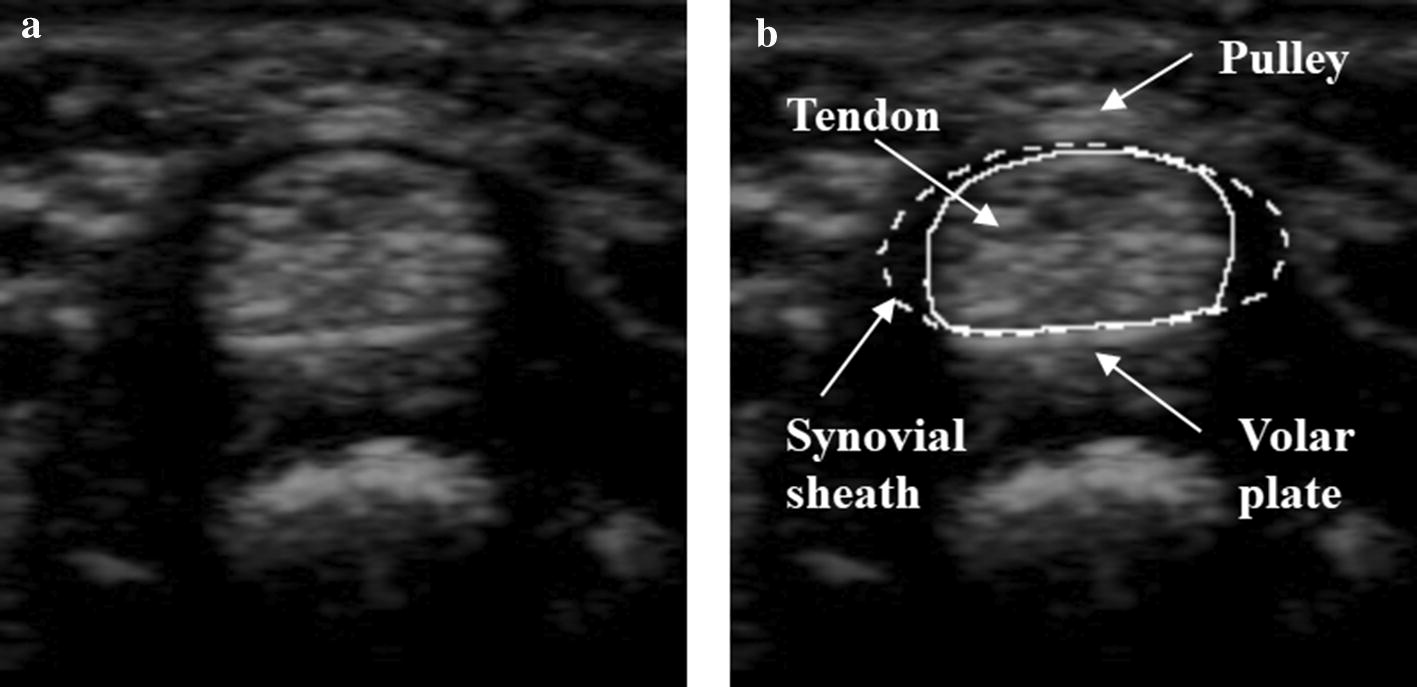
Ultrasound image of the tendon and synovial sheath of a finger. **a** Original image acquired around A1 pulley. **b** Tendon (solid line), synovial sheath (dotted line) area, and surrounding tissues

In clinical settings, ultrasound imaging is widely used to diagnose finger tissue conditions. Sato et al. [5] compared the thickness of the pulley and flexor tendon in healthy fingers and fingers associated with the trigger finger condition. Yang et al. [6] proposed a method for identifying the position and thickness of the A1 pulley. Kim et al. [7] found that the thickness of the A2 pulley and flexor tendon under the A2 pulley are related to the severity of trigger finger. Figure 1a shows an ultrasound transverse image of the finger located at the position of A1 pulley. As shown in Fig. 1b, the elliptical shape of the tendon appears above the volar plate of the finger bone and is surrounded by the synovial sheath, which is indicated by the dotted line.

In addition to diagnosis, ultrasound can also be used as part of the surgical treatment of the trigger finger condition. Ultrasound-guided minimally invasive surgery has the advantages of fast recovery and minimal tissue damage. However, a highly experienced surgeon is required to perform the operation; therefore, surgical training and planning systems are very desirable [8–11]. The anatomical target and nearby organs or tissues are present in a 3D virtual environment, of which the user can access. Typically, to achieve more realistic and accurate simulations or planning, the target models are built from images and contours of the organs or tissues must be manually outlined to construct models. In this study, to extract tissues contours, we propose a CNN-based method for automatic segmentation of the tendon and synovial sheath.

Numerous studies used automatic algorithms of image processing techniques to segment soft tissue. Gupta et al. [12] proposed an automatic segmentation method of supraspinatus tendon based on the curvelet transform. Hamameh and Gustavsson [13] proposed a method for segmentation of the human left ventricle based on a combination of active shape model (ASM) and active contour mode (ACM). Cunningham et al. [14] proposed a method for segmentation of cervical muscles and the spine, in which ASM was used to refine the segmentation. Martins et al. [15] proposed a method for segmentation of the extensor tendon in dorsal longitudinal view, which relied on an active contours’ framework. The ASM and ACM are the most widely used methods for soft tissue segmentation. In our previous work [16], we proposed an adaptive texture-based ASM (ATASM) for segmentation of the tendon and synovium sheath. The ATASM applies a shape model together with texture feature generation and then uses a genetic algorithm-based energy optimization. In our previous work, the acquired ultrasound images were separated into two groups, clear and fuzzy, based on image quality. The average dice similarity coefficient (DSC) of ATASM is 0.905 and 0.874 for tendon and sheath, respectively, which is superior to the ASM, ACM, and texture-based ASM. Although the ATASM had been demonstrated to be a powerful method for finger tissue segmentation, it struggled with complicated computations, such as locating the shape model and image grouping.

Recently, deep convolutional neural networks (CNNs) have demonstrated enormous potential in the field of medical image analysis. Unlike traditional machine learning methods, deep neural networks do not require handcrafted features, such as texture features for training, and can be trained end-to-end for object segmentation. Thus, the CNN is a suitable choice for separating the regions of the tendon and synovial sheath. In biomedical image segmentation, recent success in precise image segmentation was achieved using the encode–decode structure, such as the fully convolutional network (FCN) [17], SegNet [18], and U-Net [19]. However, the larger number of feature maps always significantly increases the scale of the network, including network depth and the size of each hidden layer. To efficiently control the number of feature maps in different channels of parallel computation, the DenseNet [20] was proposed. A newly developed CNN, called the fully convolutional DenseNet (FC-DenseNet) [21], incorporates the fully convolutional network (FCN) with the DenseNet, which has been demonstrated to be a powerful method for object segmentation. In our preliminary work [22], we attempted to apply FC-DenseNet for the segmentation of tendons from ultrasound images; however, the average dice similarity coefficient (DSC) was only 0.88 for 380 test images.

The dilated convolution [23] applied multiscale information in segmentation tasks without losing resolution. After applying this technique, the receptive field of the convolution can be exponentially expanded. Javaid et al. [24] compared the standard U-Net and dilated U-Net for multi-organ segmentation of chest CT images and found that dilated U-Net yielded the best results. Perone et al. [25] devised a model for spinal cord gray matter (GM) segmentation using deep dilated convolutions from an MRI dataset and reported that—in a GM segmentation challenge—the state-of-the-art results were more favorable in 8 out of 10 metrics. For combining this technique with the DenseNet, Zhou et al. [26] proposed an adaptive DenseNet that uses the mechanism of dilated convolution to classify and locate thoracic disease based on X-ray images of the chest. Yang et al. [27] proposed a network called DenseASPP (densely connected Atrous Spatial Pyramid Pooling) for semantic segmentation of street scenes where dilated convolution layers were applied. Although these structures achieved outstanding segmentation performance, use of dilated convolutional DenseNet for soft tissue segmentation is still very rare, to the best of our knowledge.

Deeply supervised nets [28] minimize classification error, while the learning process of hidden layers is direct and transparent. Deeply supervised nets introduce a common objective to the individual hidden layers instead of the overall loss function, and then use the layer-wise training strategy to enhance the classification capability of the feature maps. Mo et al. [29] proposed a deep-supervised FCN for segmentation of the vessel from retinal images. Chung et al. [30] proposed a dense block applied FCN with deep supervision for segmentation of the liver from CT images. Lei et al. [31] proposed a method for ultrasound prostate segmentation based on 3D V-Net with a deep supervision mechanism; this method demonstrated high accuracy, with a DSC of 0.92. These previous studies demonstrated the effectiveness of deeply supervised training for segmentation CNNs.

The dilated convolution and deep supervision are CNN techniques that can further improve the details of segmentation. However, a CNN structure that combines both of these is rarely seen. In this article, we attempt to integrate the concepts of deeply supervised net and dilated convolution into the FC-DenseNet, resulting in a new CNN termed “deeply supervised dilated FC-DenseNet (D2FC-DN)”. This work proposes a hybrid CNN technique method for finger tissue segmentation, in which the FC-DenseNet is the basic structure and dilated convolutional is combined to allow multiscale feature map information without losing resolution. Then, deeply supervised learning is applied to increase the transparency of the hidden layers to ensure that each hidden layer output is trained under supervision. In the experiments conducted in this work, this new CNN was used to segment the finger tendon and synovial sheath from transverse ultrasound images.

This paper is organized as follows. The data material of this study and experimental results are described in “Results” and “Discussion” sections that demonstrate Chuang’s dataset [16] with independent images that were used to compare the results of this study with conventional methods, and a dataset with eight groups of images used for model building. Conclusions of this study are presented subsequently. The proposed D2FC-DN is presented in “Methods” section, and the applied FC-DenseNet, dilated convolution, and deeply supervised methods are also described.

## Results

### Data materials

Two datasets of ultrasound images of fingers at the A1 pulley, captured in the transverse view, were used in this study. The first set was the Chuang’s dataset [16], which was used to make comparisons with the proposed method. The second set consisted of eight groups of images captured for building a model of finger tissue for a surgical training system, termed the modeling-building (MB) dataset. Chuang’s dataset consists of ultrasound images, of which 74 are finger tendon images and 57 are synovial sheath images. All of the images have segmentation ground truth. The images in this dataset were classified into clear and fuzzy groups, according to the difference in average intensity of two bounding boxes on the bottom boundary of the tendon in the image. For the tendon images, there are 38 images in the clear group and 36 images in the fuzzy group; for the synovial sheath images, there are 30 and 27 images in the clear and fuzzy groups, respectively. The physical spacing of the images was 0.075 × 0.075 mm^2^ for each pixel. The data were acquired using the t3000 ultrasound system (Terason, Burlington, MA, USA) with a 13-MHz probe.

**Fig. 2:**
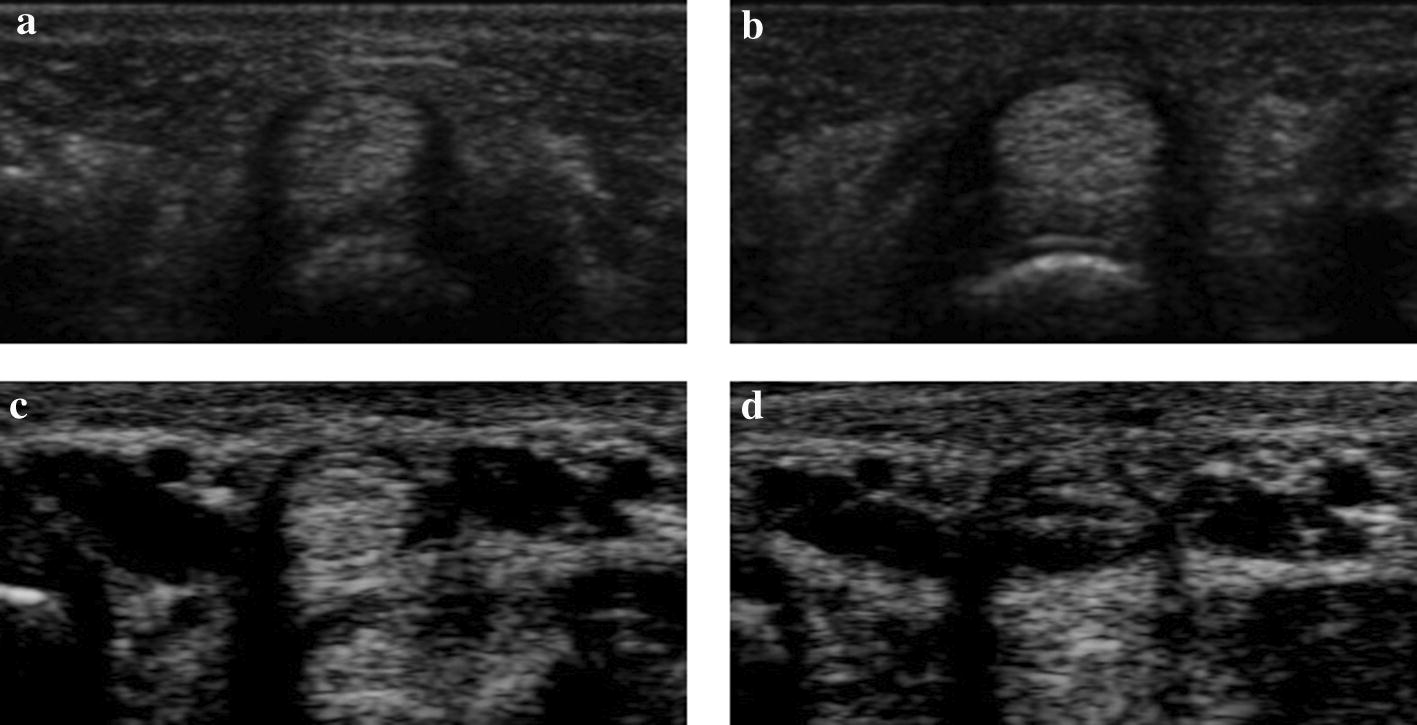
Dataset image samples. **a**, **b** Chuang’s dataset; **c**, **d** MB dataset

The MB dataset consists of eight repeated acquisitions of ultrasound images of a subject on different days, which were acquired at National Cheng Kung University Hospital (IRB number: B-ER-101-012). A total of 1035 images were acquired, with approximately 90–200 images in each group. In this dataset, the ultrasound images were captured by the Siemens ACUSON S2000 Ultrasound System, using an 18–5.5 MHz linear 18L6 HD transducer. 2D transverse ultrasound images of the right-hand’s middle finger at the A1 pulley were captured. The tendon and synovial sheath regions in the images were annotated by Dr. T. H. Yang as the ground truths. The pixel spacing of the images is 0.07 × 0.07 mm^2^ for each pixel. Figure 2 shows example images of the datasets used in this study, which have a resolution of 384 × 192 pixels. Clear and fuzzy images from Chuang’s dataset are shown in Fig. 2a, b and images from the MB dataset in Fig. 2c, d. We found four images in Chuang’s dataset that contain two finger tissues; thus, we kept the tissue of interest and masked the other one in black.

### Experiments

We first compared the proposed network with ATASM, U-Net, FC-DenseNet, and dilated FC-DenseNet (DFC-DN), using Chuang’s dataset. These methods were evaluated by fourfold cross-validations. The network was pre-trained with images from the MB dataset. Then, for finger tissue modeling, we also evaluated the accuracy of our segmentation method. There were eight groups of continuous images in the MB dataset. During evaluation, one group was left for testing and other groups for training, in which each group was tested in turns. In practice, each training group only generated one sequence of training images, more precisely, the first image of each training group was selected as the start image and the other training images were subsequently extracted from the start image at intervals of four images. The network was pre-trained with images from Chuang’s dataset.

In this study, because the number of images was limited, the number of training images was increased using data augmentation in which images were flipped, translated, and scaled. The parameters of the augmentation are shown in Table 1, in which the augmentation type, random interval of parameter and unit are shown. In this study, each image in the training group was augmented by vertical flipping, four times of random translation and four times of random rotation. After this augmentation, the amount of training images was increased to ten times. In addition, to further increase the variety of the data, the augmentation of rotation, shrinking, translation, noise adding, and gamma transformation were randomly applied to the data in every training epoch. In this study, the networks were built with similar architecture to facilitate comparisons. The number of parameters of the U-Net was 5.24 million, while FC-DenseNet, DFC-DN, and D2FC-DN were approximately 4.97 million. The running time of U-Net, FC-DenseNet, DFC-DN, and D2FC-DN were 0.02565, 0.03281, 0.03164, and 0.03151 s per image, respectively. It is worth noting that the architecture of DFC-DN and D2FC-DN were nearly identical, except for the deeply supervised part that had approximately 3000 additional parameters. The input of the networks was the ultrasound image in size of 384 × 192 × 1 and the output was the segmentation result with 384 × 192 × 1. The training strategy and parameter settings of these networks were the same.

**Table 1:** Data augmentation

Augmentation	Parameters	Unit
Flipping	[Vertical]	–
Translation^a^	$\left[ { - 32, 32} \right]$	Pixel
Scaling^a^	$\left[ {10, 50} \right]$	Pixel

^a^The parameters of horizontal and vertical are independent

**Fig. 3:**
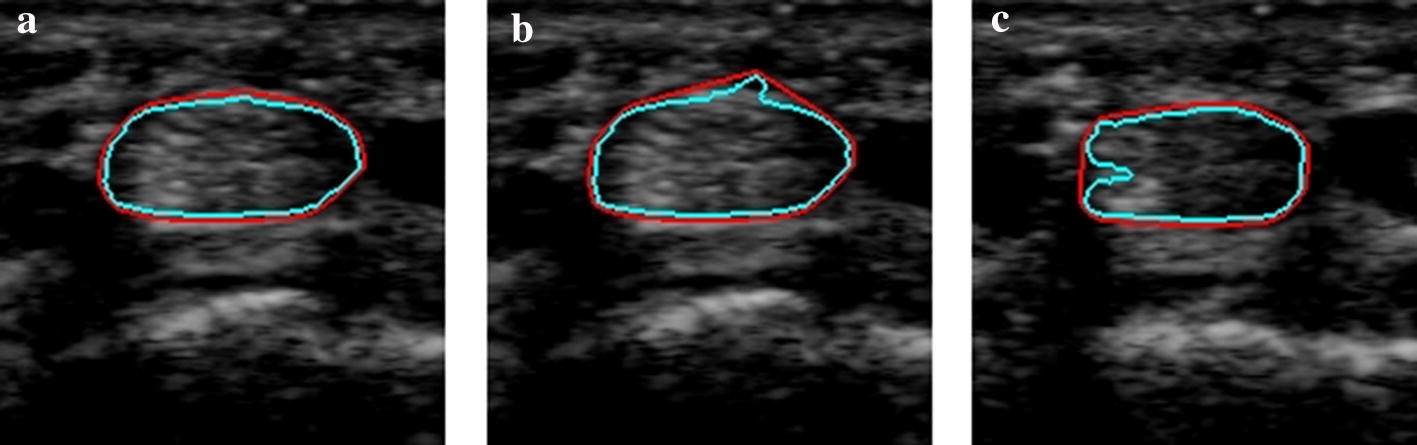
Predicted result contours (cyan) and the convex hull outputs (red). **a** Smooth contour (CHD = 1.41). **b** Contour with a bud (CHD = 7.62). **c** Contour with a groove (CHD = 18.68)

We used the DSC, mean of absolute distance (MAD), Hausdorff distance (HD) and Yasnoff [32] to evaluate the segmentation results of the methods. These measures show the similarity of region and contour between the predicted results and the ground truth. The definitions of these are shown below.

1 $${\text{DSC}}\left( {X,Y} \right) = \frac{{2\left| {X \cap Y} \right|}}{\left| X \right| + \left| Y \right|} ,$$

2 $${\text{MAD}}\left( {A,B} \right) = \frac{1}{2}\left[ {\frac{1}{\left| A \right|}\mathop \sum \limits_{a \in A} \mathop {\inf }\limits_{b \in B} d\left( {a,b} \right) + \frac{1}{\left| B \right|}\mathop \sum \limits_{b \in B} \mathop {\inf }\limits_{a \in A} d\left( {a,b} \right)} \right] ,$$

3 $${\text{HD}}\left( {A,B} \right) = { \hbox{max} }\left\{ \mathop {\sup }\limits_{a \in A} \mathop {\inf }\limits_{b \in B} d\left( {a,b} \right),\mathop {\sup }\limits_{b \in B} \mathop {\inf }\limits_{a \in A} d\left( {a,b} \right)\right\} ,$$

4 $${\text{Yasnoff}}\left( {A,B} \right) = \frac{100}{\text{W}}\sqrt {\mathop \sum \limits_{a \in A} \left( {\mathop {\hbox{min} }\limits_{b \in B} d\left( {a,b} \right)} \right)^{2} } ,$$

where $\left| \cdot \right|$ denotes the pixel number of the region $X$ (predicted result), $Y$ (ground truth) or $X \cap Y$ (overlapped region of the predicted result and ground truth); $A$ and $B$ are the contours of $X$ and $Y$, respectively; $d\left( {a,b} \right)$ is the distance between $a$ and $b$, where $a$ is a pixel on $A$, and $b$ is a pixel on $B$; W is the number of pixels in the image. To evaluate the smoothness of the output contour, a convex hull Hausdorff distance (CHD) is defined, as described below

5 $${\text{CHD}}\left( {A,C} \right) = { \hbox{max} }\left\{ \mathop {\sup }\limits_{a \in A} \mathop {\inf }\limits_{c \in C} d\left( {a,c} \right),\mathop {\sup }\limits_{c \in C} \mathop {\inf }\limits_{a \in A} d\left( {a,c} \right)\right\} ,$$

where $A$ is the contour of the predicted region, $C$ is the contour of the convex hull of the predicted region; $a$ is a pixel on $A$, and $c$ is a pixel on $C$. So, if the contour of the predicted result is smooth, CHD will be low, otherwise it is high. Figure 3 shows examples of result contours with their convex hull contours. It can be observed that the CHD increases as the smoothness of the result contours decreases. Note that the artifacts are filtered when calculating HD and CHD, because they are very sensitive to noise.

### Segmentation performance evaluation

Chuang et al. [16] proposed a conventional ultrasound image segmentation method called ATASM, which showed state-of-art results on finger tendon and synovial sheath. In this study, we used the same dataset to evaluate segmentation performance. To separate the Chuang’s dataset, we used the same criterion as Chuang’s study. The difference between the average intensities of two 15 × 15 windows, which are above and below the bottom tendon boundary, was calculated. If the average intensity of the window above is higher than the one below by 30, the image belongs to the clear group; otherwise, it belongs to the fuzzy group. The clarification of clear and fuzzy images, which depends on the boundary of the tendon, can be referred in the literature of Chuang’s dataset.

The segmentation evaluation of the tendon is shown in Table 2, where the clear group is shown on the left side and the fuzzy group is on the right. Our proposed method obtains the best DSC and outperforms ATASM, with 2% higher on the clear group. DFC-DN and D2FC-DN both have the highest DSC in the fuzzy group that is approximately 2% more than ATASM’s. For the contour similarity measurements, the proposed method achieves the best MAD, HD and Yasnoff for both the clear and fuzzy groups. In addition, D2FC-DN has the best CHD in both groups, which also indicates it can give smooth output contours. It is worth noting that ATASM can achieve better or on par results with U-Net and FC-DenseNet on DSC and better MAD than these two methods.

**Table 2:** Tendon segmentation results of Chuang’s dataset (mean ± standard deviation)

Methods	Clear					Fuzzy				
	DSC	MAD	HD	Yasnoff	CHD	DSC	MAD	HD	Yasnoff	CHD
ATASM [16]	0.91 ± 0.03	3.14 ± 1.14	–	–	–	0.90 ± 0.03	3.34 ± 0.99	–	–	–
U-Net	0.87 ± 0.14	7.93 ± 7.84	22.42 ± 33.76	0.55 ± 0.66	9.10 ± 13.17	0.87 ± 0.08	10.14 ± 8.95	28.29 ± 36.36	0.72 ± 0.71	11.88 ± 13.56
FC-DenseNet	0.91 ± 0.05	5.70 ± 6.69	18.62 ± 34.02	0.35 ± 0.54	6.73 ± 12.37	0.90 ± 0.05	4.80 ± 5.53	14.27 ± 22.77	0.26 ± 0.46	5.37 ± 10.61
DFC-DN	0.92 ± 0.03	2.74 ± 1.43	7.52 ± 3.72	0.09 ± 0.13	2.13 ± 2.60	0.92 ± 0.04	2.79 ± 1.58	7.41 ± 3.63	0.09 ± 0.09	1.81 ± 1.03
D2FC-DN	0.93 ± 0.03	2.51 ± 1.02	7.45 ± 2.82	0.07 ± 0.03	2.02 ± 2.79	0.92 ± 0.04	2.74 ± 1.36	7.36 ± 3.70	0.07 ± 0.05	1.39 ± 0.79

**Fig. 4:**
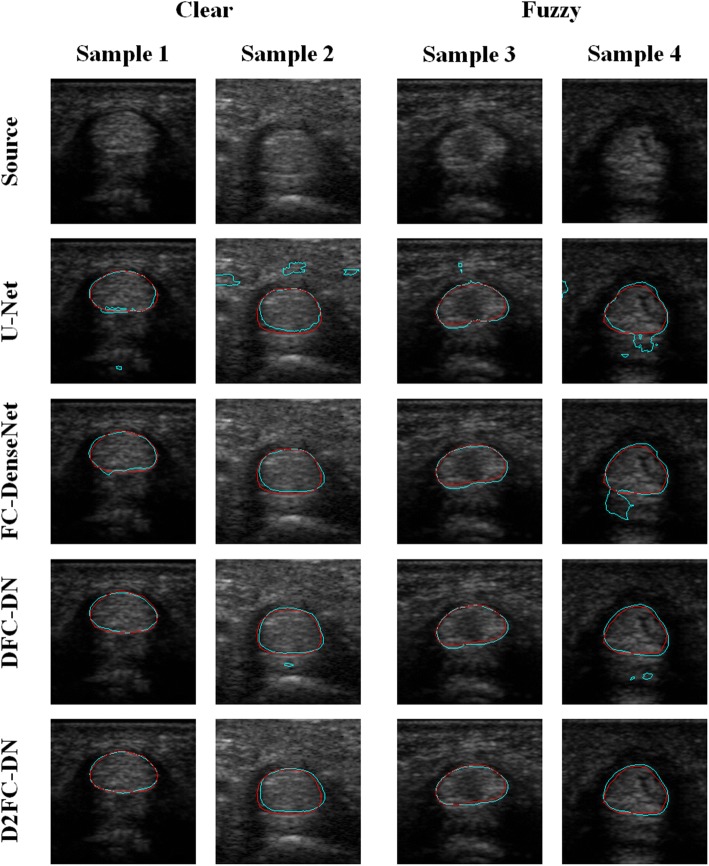
Segmentation results of tendon on Chuang’s dataset

Figure 4 shows examples of the visualization evaluation of the tendon segmentation on Chuang’s dataset. Samples 1 and 2 are in the clear group, and the other two are in the fuzzy group. The results are magnified for better visualization. The source and result images of the methods are shown from top to bottom where the predicted result contours are shown in cyan color and the ground truth are in red. The results of U-Net are over-segmented and many artifacts can also be seen. The results of FC-DenseNet are significantly better; however, sample 4 is largely over-segmented. The results of DFC-DN and D2FC-DN are quite similar, which indicates that their network structures are very similar except that there are some additional parameters for the deeply supervised training. Some little artifacts on samples 2 and 4 of DFC-DN outputs can be observed; in addition, the contours are more fitting on D2FC-DN.

Table 3 shows the synovial sheath segmentation results of the methods on Chuang’s dataset. The proposed method achieved the best DSC that is 3% higher than ATASM in both the clear and fuzzy groups. In general, the D2FC-DN outperformed the other CNN methods in MAD, HD, Yasnoff, and CHD. Comparison of the segmentation results of tendon and synovial sheath showed that the DSC difference of ATASM is large, with a 4% difference in the clear group and 2% in the fuzzy group. This indicates that the segmentation criteria are not alike between these tissues. However, the differences are smaller in CNN methods; for example, only a 1% difference in the fuzzy group for D2FC-DN. In other words, the CNN method gives more stable results with different segmentation conditions.

**Table 3:** Synovial sheath segmentation results of Chuang’s dataset (mean ± standard deviation)

Methods	Clear					Fuzzy				
	DSC	MAD	HD	Yasnoff	CHD	DSC	MAD	HD	Yasnoff	CHD
ATASM [16]	0.87 ± 0.04	5.12 ± 1.67	–	–	–	0.88 ± 0.04	4.54 ± 1.37	–	–	–
U-Net	0.87 ± 0.07	8.10 ± 6.25	26.69 ± 34.07	0.51 ± 0.57	10.27 ± 14.45	0.88 ± 0.05	8.62 ± 4.34	19.01 ± 19.46	0.64 ± 0.39	8.77 ± 8.97
FC-DenseNet	0.89 ± 0.05	5.30 ± 4.78	18.04 ± 21.51	0.24 ± 0.39	6.17 ± 9.81	0.89 ± 0.05	4.87 ± 3.64	11.39 ± 5.10	0.23 ± 0.30	3.75 ± 3.77
DFC-DN	0.90 ± 0.04	4.00 ± 1.92	12.34 ± 4.96	0.11 ± 0.06	2.78 ± 2.91	0.90 ± 0.04	3.64 ± 1.58	10.49 ± 5.62	0.10 ± 0.05	2.51 ± 2.76
D2FC-DN	0.90 ± 0.05	3.92 ± 1.74	12.38 ± 4.90	0.12 ± 0.06	2.60 ± 3.61	0.91 ± 0.04	3.29 ± 1.43	9.53 ± 3.68	0.09 ± 0.04	1.66 ± 0.87

**Fig. 5:**
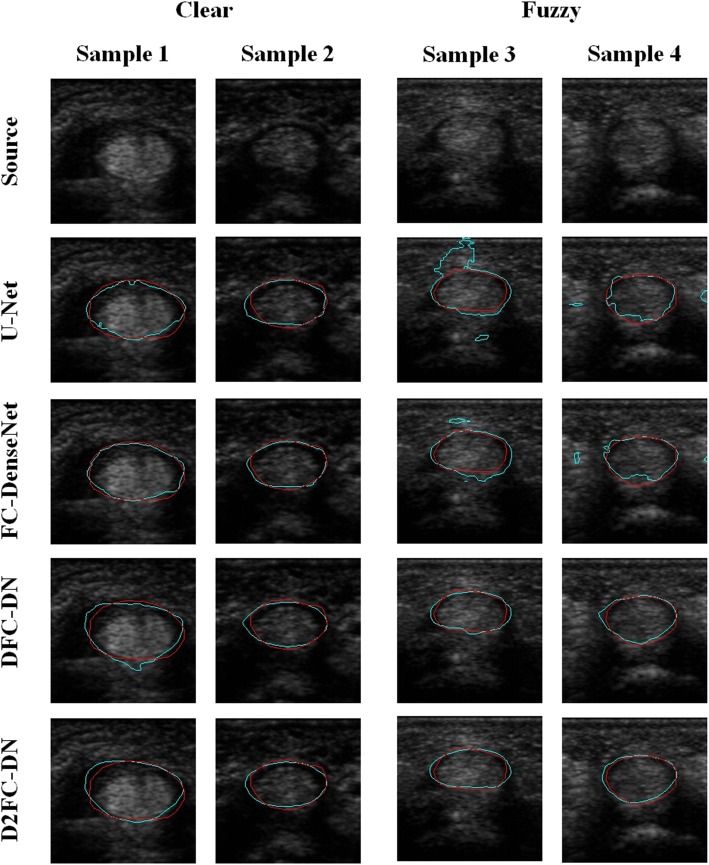
Segmentation results of synovial sheath on Chuang’s dataset

The synovial sheath visualization results are shown in Fig. 5; the arrangement of the results are the same as Fig. 4. The results of U-Net are largely over-segmented on sample 3 and irregular contours were observed on samples 1 and 3; however, the result of sample 2 is rather fitting. The result contours of FC-DenseNet are not smooth enough on the samples, over and less segmented on samples 3 and 4, respectively. Compared to FC-DenseNet, the result contours of DFC-DN are better; however, the results are over-segmented on the samples. The result contours of D2FC-DN are smooth and better fitting on the samples, although there is still little not a complete match on sample 1.

## 3D models of MB dataset

The MB dataset, which consists of eight groups of ultrasound images, was used to build 3D finger tissue models for surgical training. We first evaluated segmentation performance between the methods, then we used the segmentation results to build the 3D models for surgical training, and finally we show the segmentation accuracy of each group.

Table 4 shows the segmentation results of the methods, where the results of tendon are shown on the left side and synovial sheath on the right. The proposed method displayed the best performance on tendon. An extremely high DSC is achieved by our method of approximately 95% for synovial sheath, where DSC, MAD, and HD are on par between D2FC-DN and DFC-DN. However, CHD is significantly better on D2FC-DN, which shows the proposed method has the ability to yield smoother contours.

**Table 4:** Tendon and sheath segmentation results of MB dataset (mean ± standard deviation)

Methods	Tendon				Synovial sheath			
	DSC	MAD	HD	CHD	DSC	MAD	HD	CHD
U-Net	0.87 ± 0.10	4.71 ± 3.51	12.43 ± 8.51	7.24 ± 6.77	0.91 ± 0.07	4.14 ± 4.07	12.54 ± 11.03	8.87 ± 9.13
FC-DenseNet	0.90 ± 0.06	3.61 ± 2.92	9.56 ± 9.32	3.16 ± 5.57	0.94 ± 0.05	2.21 ± 2.10	8.03 ± 9.37	3.47 ± 6.22
DFC-DN	0.91 ± 0.04	2.82 ± 1.53	7.79 ± 3.33	1.92 ± 1.55	0.95 ± 0.02	1.95 ± 1.01	6.56 ± 3.07	2.14 ± 3.01
D2FC-DN	0.92 ± 0.04	2.67 ± 1.38	7.62 ± 3.21	1.53 ± 0.84	0.95 ± 0.03	1.93 ± 1.02	6.63 ± 2.79	1.61 ± 1.41

**Fig. 6:**
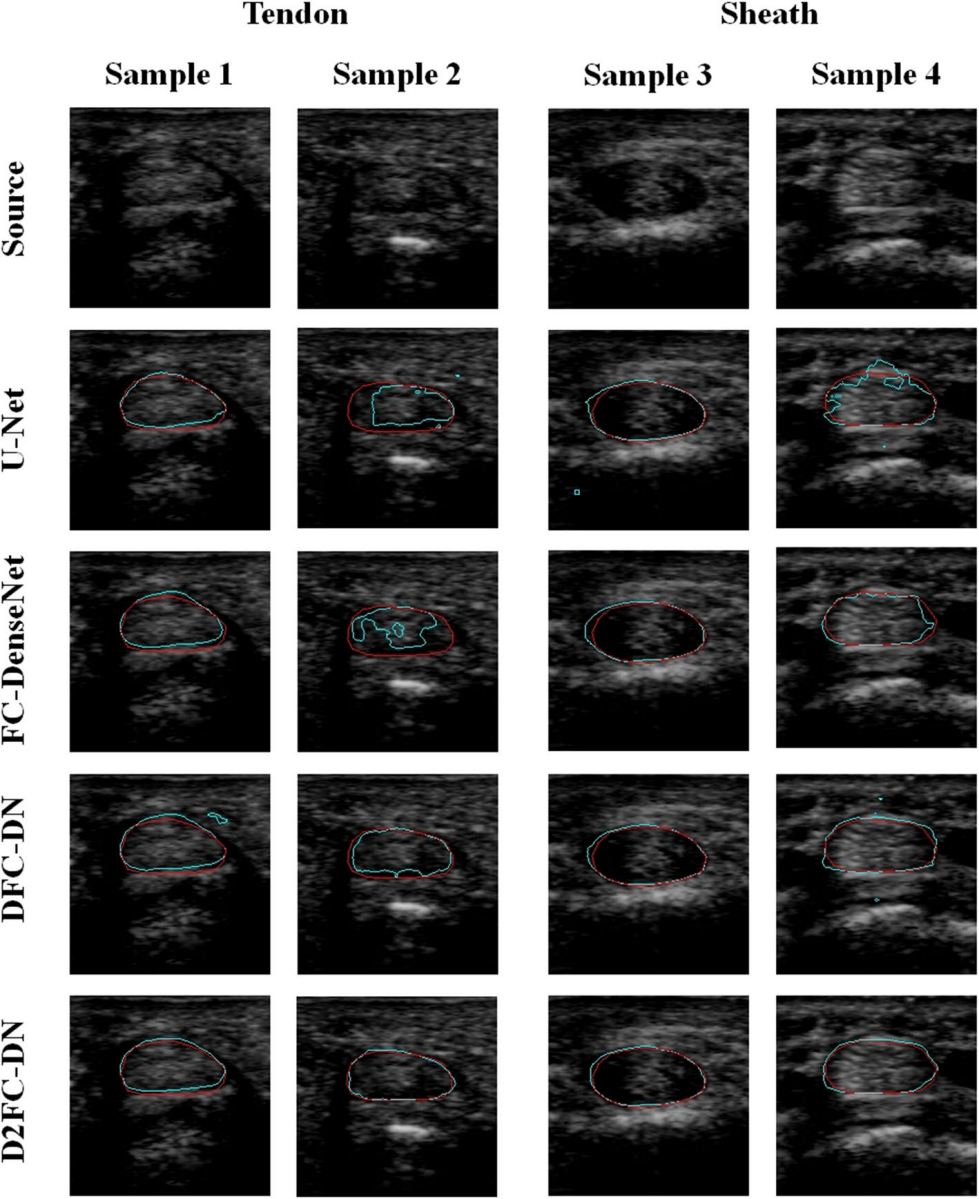
Segmentation results of tendon and synovial sheath images

Figure 6 shows the visualization results of the CNN methods; the first two samples are tendon and the other two are synovial sheath. The results are magnified for better visualization. The result contours of the methods are shown from top to bottom in cyan color, and the ground truth is in red. The U-Net results are less segmented for samples 2 and 4, and the contours of samples 1 and 3 are not smooth enough. The results of FC-DenseNet and DFC-DN are similar, but a large false segment on sample 2 resulting from the FC-DenseNet method and small artifacts on samples 1 and 4 from DFC-DN exist. It can be observed that the D2FC-DN method gives finely matching and smooth results between these methods, although the last sample is not smooth enough but it still fits.

D2FC-DN had the best segmentation performance; thus, we used this method to segment the tendon and synovial sheath of the MB dataset images. The segmentation accuracy of the eight groups is shown in Table 5. The tendon results are shown on the left side and the synovial sheath results on the right side. For the tendons, the DSC of group 1 and 8 is remarkably high and MAD is very low, which shows the similarity between the results and that the ground truth is very high. The DSC of groups 3, 4, and 6 is also high (0.92) and MAD is approximately 2.5. The DSC of the remaining three groups are approximately 0.9 and MAD is approximately 3.3. For the synovial sheaths, the DSC of all groups is amazingly high and MAD is extremely low, which may have been because of the higher consistency of the synovial sheath contour as compared to tendon on the ultrasound images. It is worth noting the variance of the HD results; however, the CHD is very small, which shows that the result contours are smooth. The increase of CHD of group 8 synovial sheath in which a few images have the problem is shown in Fig. 3c; in other words, it shows that CHD is a powerful measurement for the smoothness of a contour.

**Table 5:** Tendon and sheath segmentation results of each group in MB dataset (mean ± standard deviation)

Group#	Tendon				Synovial sheath			
	DSC	MAD	HD	CHD	DSC	MAD	HD	CHD
1	0.94 ± 0.02	1.92 ± 0.74	5.74 ± 1.81	1.16 ± 0.35	0.95 ± 0.01	1.72 ± 0.50	6.33 ± 1.99	1.35 ± 0.50
2	0.90 ± 0.04	3.25 ± 1.42	8.25 ± 3.07	1.63 ± 1.03	0.93 ± 0.05	2.50 ± 1.76	7.74 ± 3.18	1.71 ± 1.11
3	0.92 ± 0.03	2.60 ± 1.11	8.48 ± 2.26	1.71 ± 0.94	0.96 ± 0.02	1.54 ± 0.61	6.36 ± 2.43	1.83 ± 0.69
4	0.92 ± 0.03	2.46 ± 0.94	6.98 ± 2.22	1.40 ± 0.51	0.94 ± 0.02	1.95 ± 0.80	6.92 ± 2.83	1.29 ± 0.41
5	0.89 ± 0.04	3.34 ± 1.44	8.36 ± 3.37	1.77 ± 1.23	0.93 ± 0.03	2.71 ± 1.47	7.12 ± 4.51	2.28 ± 2.86
6	0.92 ± 0.04	2.52 ± 1.28	7.48 ± 3.01	1.40 ± 0.67	0.95 ± 0.01	1.72 ± 0.55	6.54 ± 2.21	1.26 ± 0.42
7	0.90 ± 0.06	3.36 ± 2.10	9.33 ± 4.98	1.34 ± 0.47	0.95 ± 0.02	1.83 ± 0.73	6.23 ± 1.90	1.21 ± 0.37
8	0.93 ± 0.02	2.13 ± 0.60	6.41 ± 2.08	2.27 ± 1.17	0.95 ± 0.03	1.84 ± 1.20	5.94 ± 3.19	2.93 ± 2.87

**Fig. 7:**
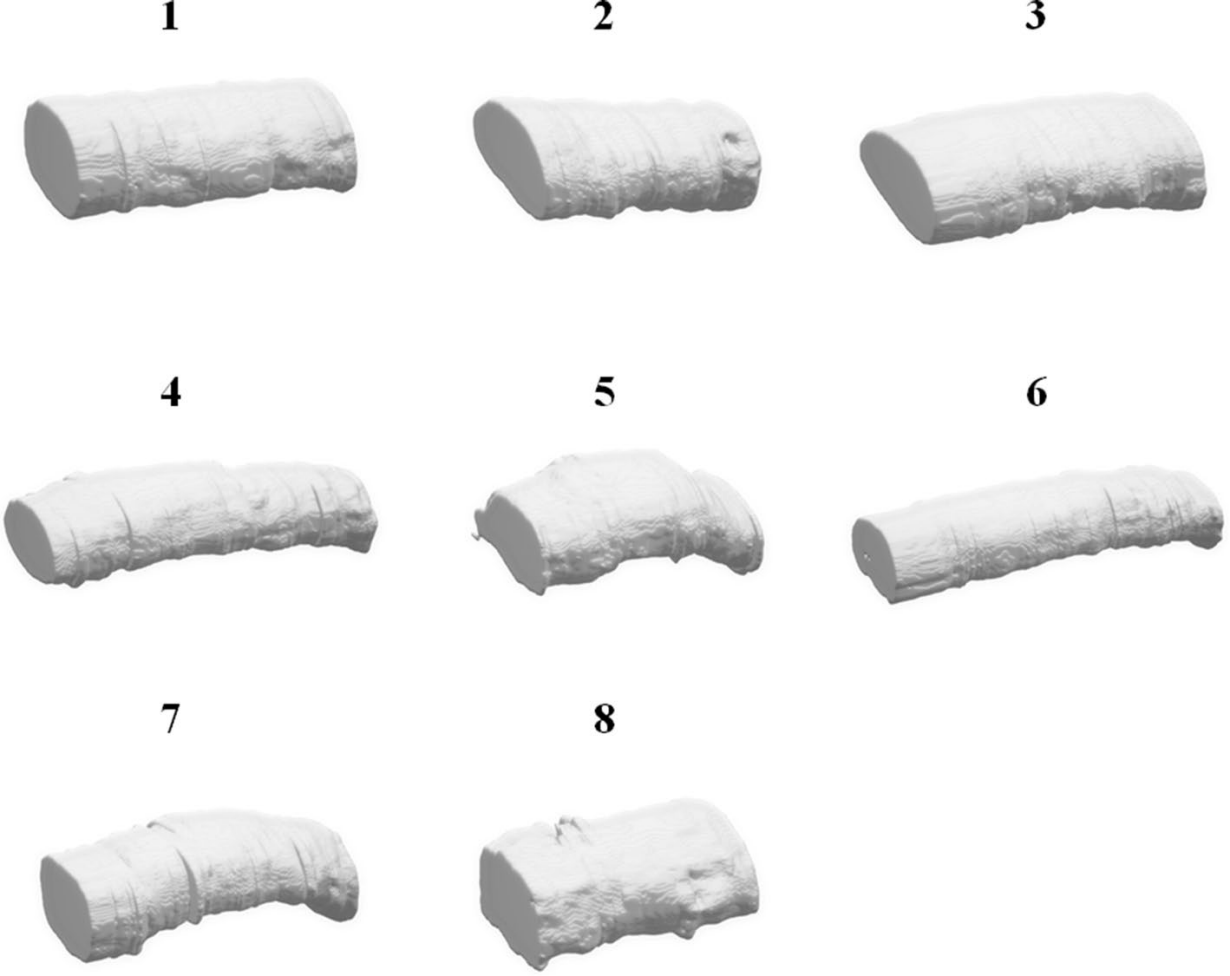
3D tendon model of MB dataset

**Fig. 8:**
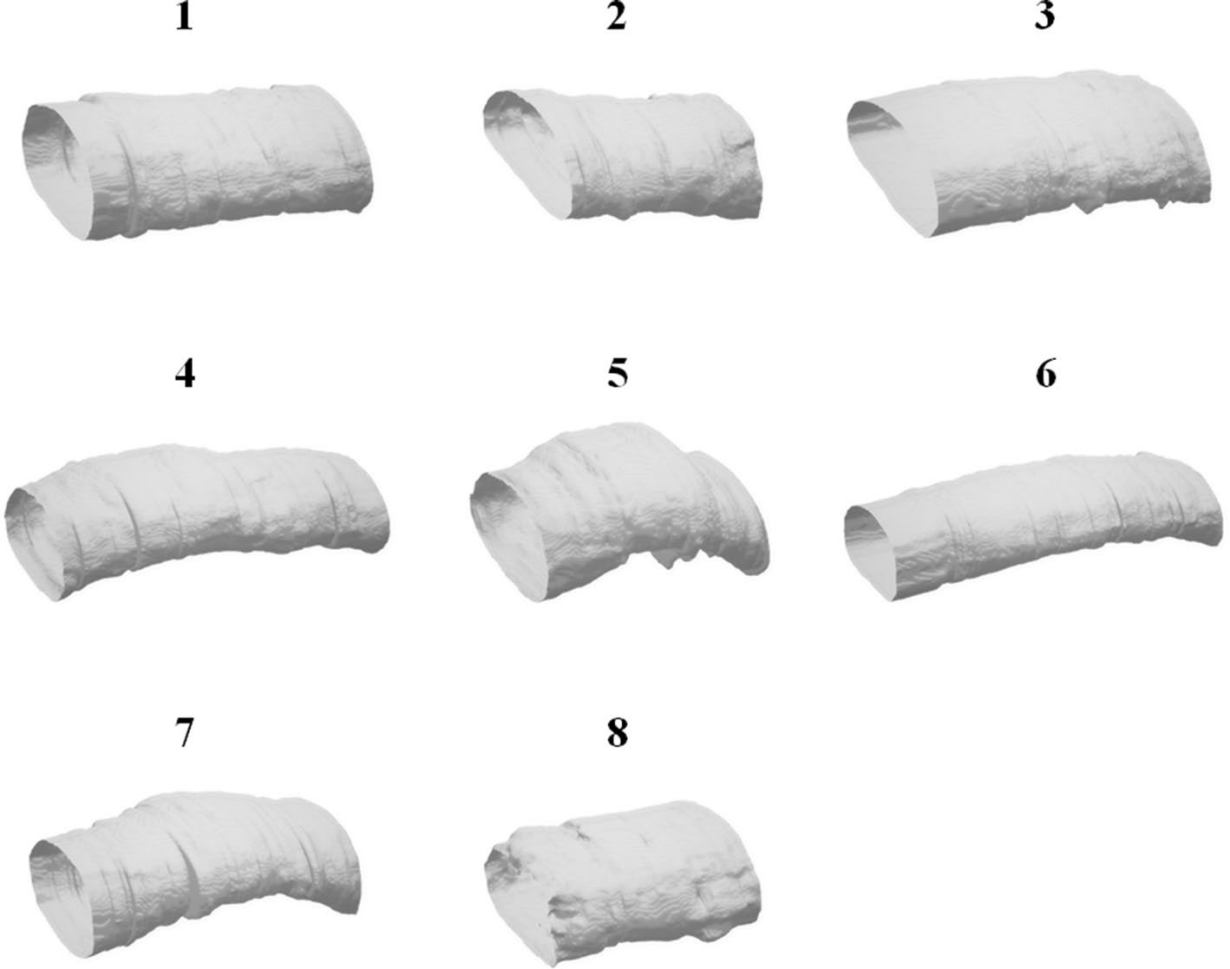
3D synovial sheath model of MB dataset

The 3D models built from the segmentation results using our proposed method are shown in Fig. 7. Groups 1 to 8 are shown, from top to bottom and left to right. Different appearances can be seen in these soft tissue models. In addition, the length of them are related to the number of acquired images, in which groups 4 and 6 are longer than the others, and groups 2 and 8 are short. Figure 8 shows the synovial sheath models; the appearances of these are thicker than the tendons. The models were built from the segmentation results using Marching cubes algorithm [33] with smoothing. To provide valid models for virtual surgical training, the artifacts outside the target tissues were removed.

## Discussion

### Advantages of D2FC-DN

For the evaluation of tendon and synovial sheath segmentations, the proposed D2FC-DN got the best performance when comparing to the other CNN methods. D2FC-DN outperforms the other deep models because it combines several effective CNN techniques to achieve their advantages and improve the segmentation results. It basically follows the encode–decode structure of the U-Net with skip connections in the network which can retrieve the lost information on the encoder side after downsampling. The dense block which is a major component of the FC-DenseNet is applied in the proposed network to achieve multiple feature map layers for convolution, which also improves the efficiency of the feature propagation. Dilated filtering is also applied to increase the receptive fields of convolution without reducing the feature resolution or increasing the filter size. Eventually, we apply the feedback supervision on each level of network output when training, so that the discriminative ability of the entire network can be improved. Due to these advantages, the proposed method not only acquires high segmentation accuracy, but also achieves the resulting contours more precisely.

### Performance of DFC-DN and D2FC-DN

**Fig. 9:**
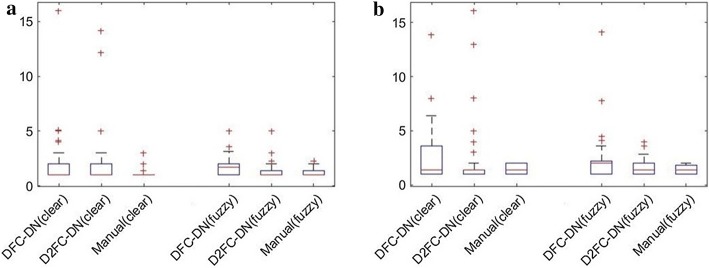
CHD results of Chuang’s dataset. **a** Tendon, **b** synovial sheath

**Fig. 10:**
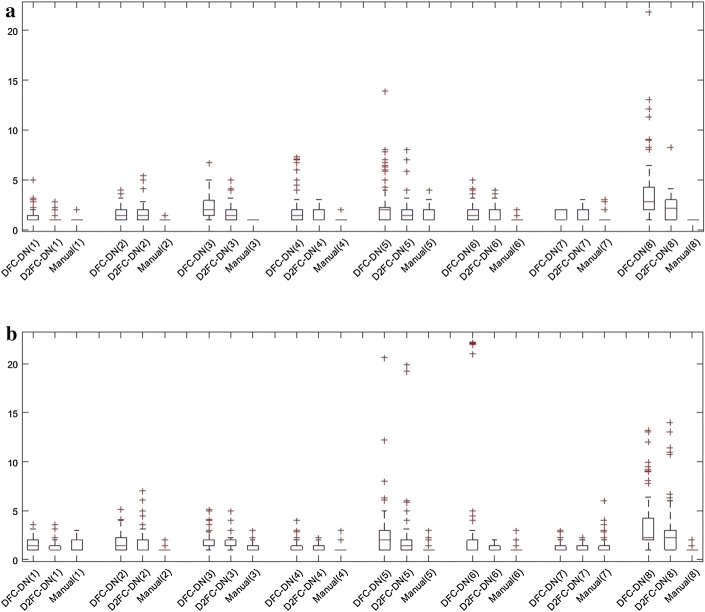
CHD results of MB dataset. **a** Tendon, **b** synovial sheath. *Method (group#)

The main network architectures of DFC-DN and D2FC-DN are similar, except the deeply supervised connections on the upsampling side for D2FC-DN. Similar performance was also observed between DFC-DN and D2FC-DN. We further explored their differences in terms of CHD, which represents the smoothness of the result contours. We also calculated the CHD of the finger tissue contours drawn by Dr. T. H. Yang and we found that they are extremely low and contain very few outliners. Figure 9 shows the CHD boxplot results of Chuang’s dataset from DFC-DN, D2FC-DN, and manual evaluation (by Dr. T. H. Yang), and the resulting distributions are discussed. For the boxplot of data, the median and the interquartile range (the 75th and the 25th percentiles) are indicated by the central mark, top and bottom edges of the box, respectively. The whiskers extend to show the extreme observations of the data, and the outliers are indicated individually by a ‘+’ symbol. It can be observed that the CHD median and interquartile range of D2FC-DN are better than DFC-DN on the fuzzy group for both tendon and synovial sheath, and they are closer to the manual ones. The CHD median and interquartile range of D2FC-DN are on par with DFC-DN on the clear groups. Figure 10 shows the CHD statistical performance of the MB dataset. It can be observed that the manual results are extremely low, which means that the result contours are smooth. The CHD median and interquartile range of D2FC-DN are mostly better than DFC-DN and closer to the manual ones. These results show that our deeply supervised strategy gives more stable, smoother contour results, in which the manual ground truth tends to have smooth contours. The visualization of the smoothness comparison between these methods can be referenced in Figs. 4, 5 and 6; for example, the contours of D2FC-DN are more smooth than DFC-DN in Fig. 5 sample 1 and Fig. 6 sample 2. Note that the artifacts are filtered for evaluation because CHD is very sensitive to noise.

### Comparison to manual segmentation results

We compared the proposed method to the manual segmentation results from Chuang’s study [16]. In the study, two users were trained to outline the tendon from 16 ultrasound images. Both of them were blinded to the ground truth and the automatic segmentation results. The average DSCs of users 1 and 2 are 0.88 ± 0.07, 0.93 ± 0.05, respectively, and the average DSC of the proposed D2FC-DN is 0.91 ± 0.04. It can be observed that user 2′s and our segmentation results are closer to the ground truth with high DSC values. User 1 has a relatively lower DSC value because two cases of the bottom areas of tendon were misjudged. We believe our proposed segmentation method can give accurate enough and reliable results when comparing with the regular and more experienced users.

For the training and testing performance of the proposed method, the training time for the Chuang’s dataset is approximately 2 h and approximately 3.5 h for the MB dataset. The segmentation result prediction time is approximately 0.03 s per image. The system was run on a PC with Intel i7 CPU, 24G RAM memory, and an NVIDIA GeForce RTX 2080 Ti display card equipped. The proposed network is implemented in Python with Tensorflow framework.

## Conclusions

This article proposed a deeply supervised dilated FC-DenseNet (D2FC-DN) for finger tendon and synovial sheath segmentation from ultrasound images. In the D2FC-DN, dilated dense block and deeply supervised training were adopted to successfully improve the contour smoothness. The proposed method achieves better results than not only the state-of-art conventional image processing method, but also certain representative CNN methods. The proposed method is fully end-to-end trained and tested; thus, it overcomes the preprocessing requirement of the conventional methods. The segmentation results were remarkably high on DSC values and had the smallest MAD of two datasets. The proposed method was applied to segment multiple groups of tendon and synovial sheath ultrasound images. The results had very high DSC and low MAD and were used to build 3D models for virtual surgical training. In the future, we will obtain more data for evaluation and migrate to the handling of soft tissue images for the surgical training system and clinical evaluation.

## Methods

### U-Net

**Fig. 11:**
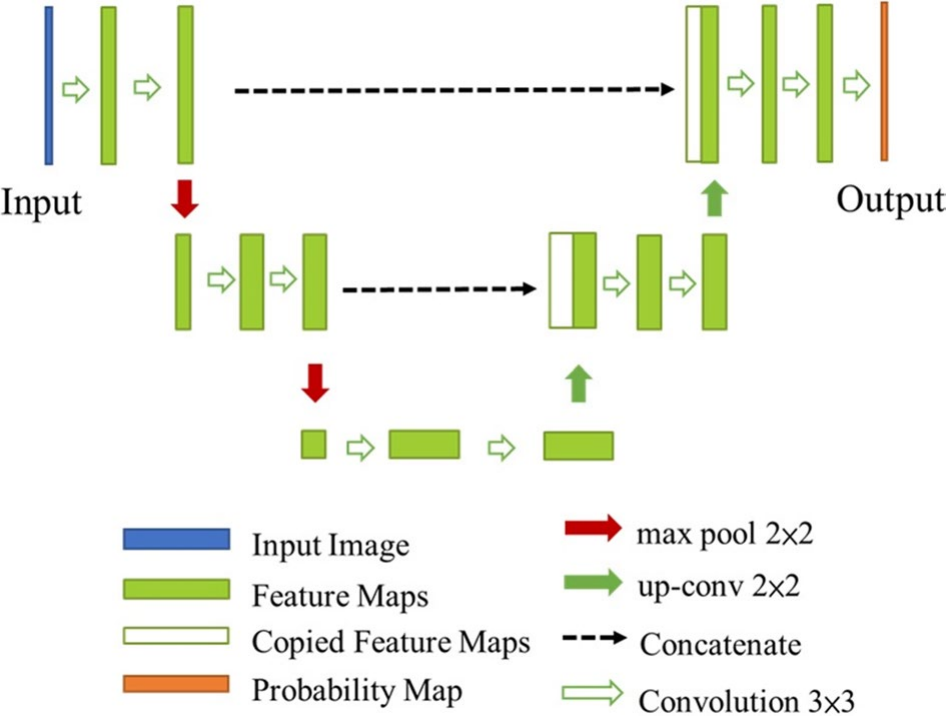
The architecture of U-Net

The U-Net [19] is a popular and basic segmentation network for medical images, which shows remarkable performance. It is a U-shape structure CNN with encoder and decoder parts, in which skip connections exist between them so that the information lost through encoding can be retrieved during decoding. Figure 11 shows an example of a U-Net with skip connections of the feature map from the downsampling side (encoder) to the upsampling side (decoder). In the network, feature map convolutions at each level are processed twice, the corresponding feature map on the downsampling side concatenates the upsampled feature map, and the result becomes an input of the convolution in the upsampling side. Downsampling and upsampling are performed by max pooling and up-convolution, respectively. According to this network, the final output map becomes the segmentation result of the input image.

### Fully Convolution DenseNet

**Fig. 12:**
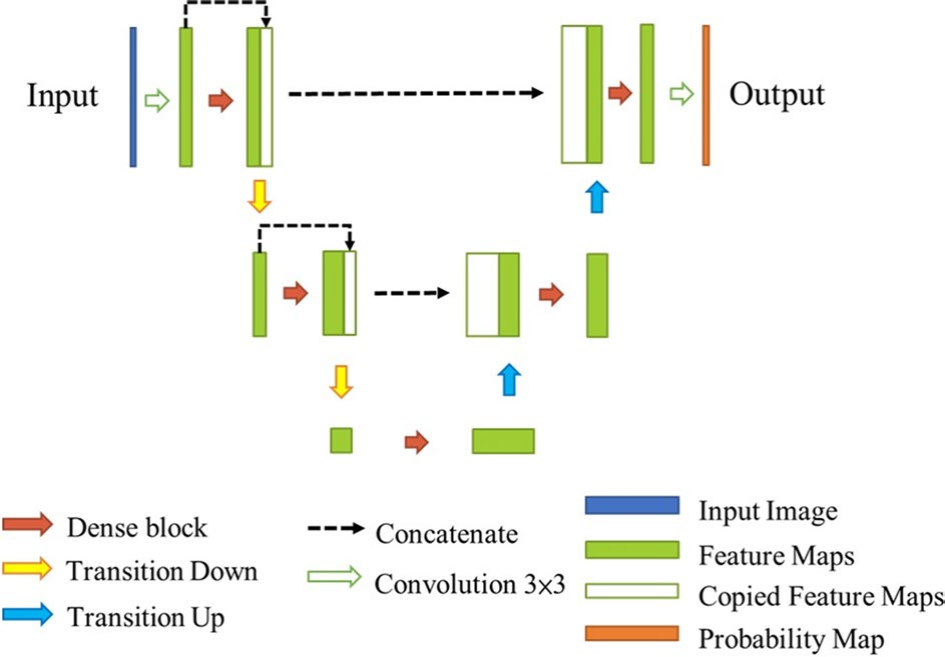
The architecture of FC-DenseNet

The DenseNet [20] was proposed to improve classification accuracy by efficiently reusing feature maps. The FC-DenseNet [21] is the extension of DenseNet to the segmentation task. FC-DenseNet not only retains the efficiency of feature reuse, but also achieves excellent performance in semantic segmentation. Figure 12 shows an example of an FC-DenseNet, which follows the U-shape structure of U-Net with the convolution replaced by the dense block of DenseNet. Furthermore, the input feature map concatenates with the output of dense block in the structure. Downsampling and upsampling are performed by the transition down- and up-layers, respectively. For a transition down-layer, a sequence of processes is applied, consisting of batch normalization, ReLU, 1 × 1 filter size convolution, and 2 × 2 max pooling with stride 2. For a transition up-layer, the corresponding sequence of processes is batch normalization, ReLU, and 3 × 3 transposed convolution with stride 2.

**Fig. 13:**
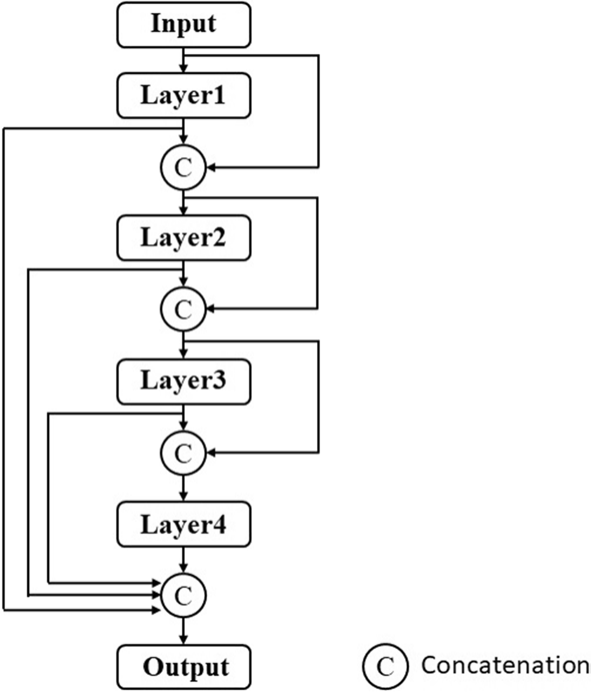
A dense block with four layers

For the design of DenseNet and FC-DenseNet, the dense block is the most important component and efficiently uses multiple feature maps. The diagram of a four-layer dense block is shown in Fig. 13, in which the output feature map of each layer is k channel, where *k* is called the growth rate. Each layer includes a sequence of processes, consisting of batch normalization, ReLU, and *n* × *n* convolution. Concatenation of the input and output of each layer becomes the input to the next layer, with the exception of the output of the final layer, which concatenates with all the outputs of the previous layers in the block. Thus, in Fig. 13, if *k* = 4, the output channel number of the block is 16. This design can prevent an increase in the number of channels when the network is deep and allows the efficient use of multiple features.

### Dilated FC-DenseNet

**Fig. 14:**
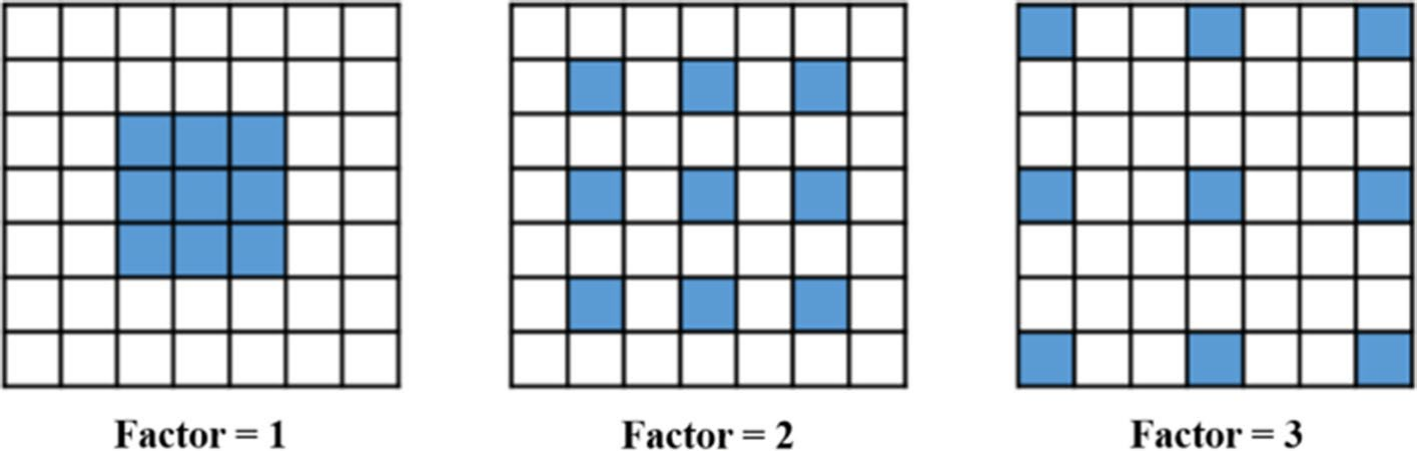
Dilated convolution with different dilation factors

Max-pooling is applied in conventional CNNs; otherwise, the filter size must be enlarged to increase the receptive field of the feature maps. However, max-pooling results in information loss, while filter size enlargement increases the computation cost. To overcome these disadvantages, dilated convolution [23] for dense prediction was proposed, where the receptive field was increased in an efficient manner. The discrete convolution of a discrete function *F* and a kernel $k$ is shown in Eq. (6),

6 $$\left( {F*k} \right)\left( p \right) = \mathop \sum \limits_{s + t = p} F\left( s \right)k\left( t \right),$$

where $p$ is the position variable and $*$ is the discrete convolution operator, in which $F :{\mathbb{Z}}^{2} \to {\mathbb{R}}$ and $k :\varOmega_{r} \to {\mathbb{R}}$ where $\varOmega_{r} = \left[ { - r,r} \right]^{2} \cap {\mathbb{Z}}^{2}$. For instance, $\varOmega_{r}$ = {(0,0), (0,1), (1,0), (1,1), (0,−1), (−1,0), (−1,−1)}, when $r = 1$. The kernel $k$ is a discrete filter with size (2 $r$ + 1)^2^. The discrete dilated convolution of the function *F* and the kernel $k$ are shown in Eq. (7),

7 $$\left( {F*_{l} k} \right)\left( p \right) = \mathop \sum \limits_{s + lt = p} F\left( s \right)k\left( t \right),$$

where $*_{l}$ is the dilated convolution operator with dilation factor $l$. The definitions of the parameters $p,s$, and $t$ are the same as in Eq. (6). Figure 14 shows multiple kernels with different dilated factors, and the dilated filter in the blue color is overlapped on a 7 × 7 receptive field of *F*. Only the overlapped position contributes to convolution and the receptive field of convolution increases when the factor increases. When the dilation factor = 1, it is equivalent to the conventional discrete convolution.

**Fig. 15:**
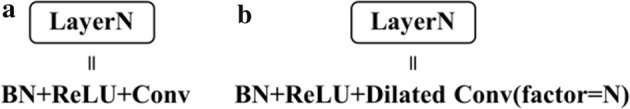
Original and dilated dense block layers. **a** Original, **b** dilated

To increase the receptive field without losing information and increasing computation cost, recent studies [23–27] applied dilated convolution layers on the networks for classification and segmentation and obtained outstanding improvements. In this study, dilated convolution was applied on the dense block to increase the receptive field, which achieved better segmentation results. Figure 15 shows the original and the dilated dense block layers. For the original dense block layer, the process sequence was batch normalization, ReLU, and (2*r* + 1)^2^ filter size convolution, where the receptive field was fixed for all layers. For the dilated dense block layer, the process sequence was the same, but dilated convolution was applied. In this study, we set *r* = 1 to apply 3 × 3 convolutions with different dilation factors. The dilation factor increased according to the layer number *N*; i.e., the dilated factor for the first layer was 1, the dilated factor for the second layer was 2, and so forth. In the dilated dense block, the convolution receptive field was concurrently enlarged on deeper layers. It should be noted that dilated convolution has the same number of parameters as the fixed style, indicating that no extra effort is required when using it.

### Deeply supervised dilated FC-DenseNet (D2FC-DN)

The layer-wise segmentation CNN consists of deep downsampling of feature maps, which may cause the gradient to vanish at low resolution levels, which affects training and network performance. To overcome these problems, deeply supervised methods [28–31] could be applied, which increase the discriminative capability and prevent the gradient from vanishing for a deep segmentation network. Because of these advantages, we applied deeply supervised learning to the segmentation network. To achieve deep supervision, pixel-based auxiliary classifiers are applied at certain intermediate layers of the network when training.

For the segmentation of finger tendon and synovial sheath, although the U-Net and FC-DenseNet showed effective segmentation performance, however, the stability of them is not enough because of over-segmentations or irregularly segmented contours. The dilated convolution has the ability to increase the receptive fields of feature abstraction so that it can overcome the disadvantages of over-segmentations; however, the resulting contour is still not smooth enough. After applying the deeply supervised strategy, we found that the smoothness of the segmentation contour was significantly improved. Consequently, the proposed method also outperformed the other networks based on the evaluation of CHD measure which was most widely used to evaluate the smoothness of contour.

**Fig. 16:**
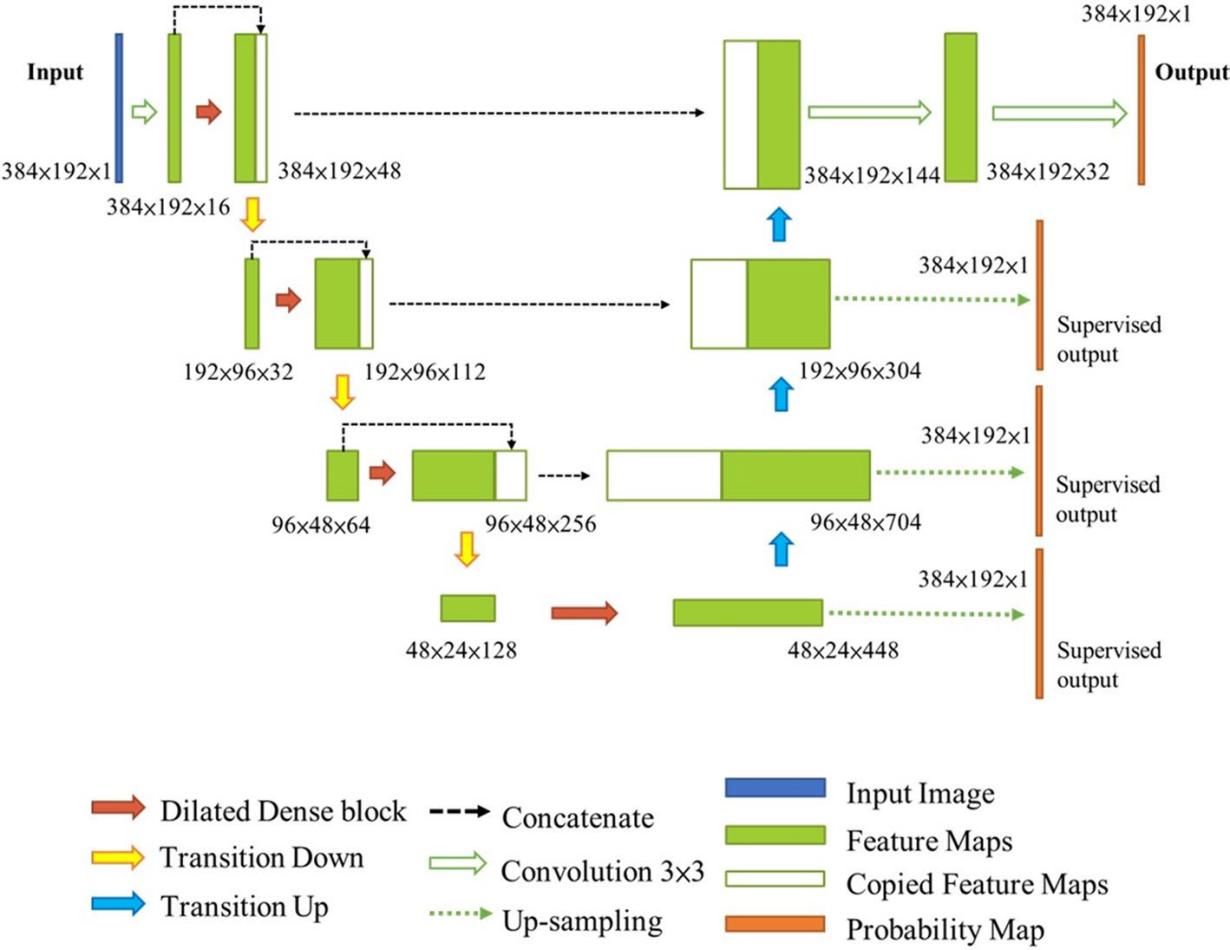
Proposed segmentation network

Figure 16 shows the proposed deeply supervised dilated FC-DenseNet (D2FC-DN) for the segmentation of tendons and synovial sheaths from ultrasound images. In this study, the network structures for the segmentation of tendon and synovial sheath were identical but were trained individually. The input was the ultrasound image, while the output was the segmentation result. The network has an encoder and decoder structure with skip connections of three levels, where the encoder and decoder are implemented by downsampling and upsampling of feature maps, respectively, and the skip connection is the corresponding concatenation of the feature maps from the downsampling side to the upsampling side. The dilated dense block is the dense block with dilated convolution layers as shown in Fig. 15. Downsampling and upsampling correspond to the transition down- and up-layers, respectively, and were the same as those of the previously mentioned FC-DenseNet. The last feature map at each intermediate level on the upsampling side was convoluted by a 1 × 1 filter to become a probability map, and then was upsampled by bilinear interpolation to the input image resolution. After thresholding on these upsampled probability maps, the binary supervision results were obtained and compared with the ground truth, which provided deeply supervised training. In this study, the binary threshold values of probability maps were all set to 0.5 to achieve the intermediate and final segmentation results.

The detailed architecture of the D2FC-DN is shown in Table 6, which corresponds to Fig. 16, in which [.] denotes a dilated dense block layer. Similar to [34], the number of layers, $n_{l}$, and the growth rate, *k*, for each level were set in an increasing manner. In this study, we set $n_{l}$ = 4 at the highest resolution level, which was increased by 1 for each downsampling. The growth rate of the network began at 8 and doubled after each downsampling; the detail settings are shown in Table 6. It is worth noting that sufficient improvement was not found when using dense convolution on the upsampling side; hence, it is done by transition up-layer to reduce the parameter size of the network.

**Table 6:** Network architecture

Layers	Output size	D2FC-DN
Convolution	$384 \times 192 \times 16$	$7 \times 7 {\text{conv}}$
Dilated dense block	$384 \times 192 \times 32$	$\left[ {3 \times 3 \,{\text{dilated conv}}} \right] \times 4, {\text{k}} = 8$
Transition down	$192 \times 96 \times 32$	$1 \times 1 {\text{conv}}$ $2 \times 2 {\text{max pooling, stride 2}}$
Dilated dense block	$192 \times 96 \times 80$	$\left[ {3 \times 3 \,{\text{dilated conv}}} \right] \times 5, k = 16$
Transition down	$96 \times 48 \times 64$	$1 \times 1 {\text{conv}}$ $2 \times 2 {\text{max pooling, stride 2}}$
Dilated dense block	$96 \times 48 \times 192$	$\left[ {3 \times 3 \,{\text{dilated conv}}} \right] \times 6, k = 32$
Transition down	$48 \times 24 \times 128$	$1 \times 1 \,{\text{conv}}$ $2 \times 2 {\text{max pooling, stride 2}}$
Dilated dense block	$48 \times 24 \times 448$	$\left[ {3 \times 3 \,{\text{dilated conv}}} \right] \times 7, k = 64$
Transition up	$96 \times 48 \times 448$	$3 \times 3\, {\text{transposed conv, stride 2}}$
Transition up	$192 \times 96 \times 192$	$3 \times 3\, {\text{transposed conv, stride 2}}$
Transition up	$384 \times 192 \times 96$	$3 \times 3\, {\text{transposed conv, stride 2}}$
Convolution	$384 \times 192 \times 32$	$3 \times 3 \,{\text{conv}}$
Convolution	$384 \times 192 \times 1$	$3 \times 3 \,{\text{conv}}$

### Training of D2FC-DN

During training, the network was initialized by Gaussian distribution with adaptive standard deviation [35]; the standard deviation is shown below

8 $${\text{std}}\,{\text{dev}} = \sqrt {\frac{2}{{\left( {2r + 1} \right)^{2} \times c_{\text{in}} }}} ,$$

where $\left( {2r + 1} \right)^{2}$ is the filter size and $c_{\text{in}}$ the channel number of the feature map. The network was optimized by the RMSProp optimizer, the size of the mini-batch was four, and the initial learning rate was $5 \times 10^{ - 4}$. Similar to [36], a polynomial decay was applied as shown below

9 $$l_{n} = l_{0} \times \left( {1 - \frac{n}{{N_{e} }}} \right)^{\alpha } ,$$

where $l_{n}$ is the learning rate at the $n$th epoch, $l_{0}$ the initial learning rate, and $N_{e}$ the stopping epoch. We set $\alpha = 1.5$, $N_{e}$ = 50 for Chuang’s dataset and $N_{e}$ = 20 for MB dataset in this study. The total loss applied to optimize the network is shown below

10 $$L_{\text{total}} = {\text{DSC}}_{\text{loss}} \left( {{\text{output}}_{\text{main}} } \right) + \sum w_{i} {\text{DSC}}_{\text{loss}} \left( {{\text{Output}}_{{{\text{supervised}}_{\text{i}} }} } \right),$$

where ${\text{output}}_{\text{main}}$ is the final output of the network, ${\text{Output}}_{{{\text{supervised}}_{\text{i}} }}$ is the supervised outputs at different levels of the network, and $w_{i}$ is the weighting for supervised levels. As explained in [37], the weighting is low at the low-resolution supervising level. We observed that the best performance was achieved when $w_{i} = \left\{ {\frac{1}{16},\frac{1}{8},\frac{1}{4} } \right\}, i = \left\{ {1,2,3} \right\}$ in terms of the evaluation measurements; thus, we adopted this setting for this study. The definition of ${\text{DSC}}_{\text{loss}}$ [38] is shown below

11 $${\text{DSC}}_{\text{loss}} = \frac{{2\mathop \sum \nolimits_{i}^{N} p_{i} q_{i} }}{{\mathop \sum \nolimits_{i}^{N} p_{i}^{2} + \mathop \sum \nolimits_{i}^{N} q_{i}^{2} }} ,$$

where *p*, *q* are the corresponding pixel class label of the predicted result and the ground truth, respectively. *N* is the total number of pixels of the image. The class label of object was set to 1 and background was set to 0. In this study, two identical networks for segmenting the tendon and synovial sheath were trained individually, using the same training strategies.

## Data Availability

The datasets used and/or analyzed during the current study are available from the corresponding author on reasonable request and with permission of the Institutional Review Board (IRB).

## Abbreviations

D2FC-DN: Deeply supervised dilated fully convolutional DenseNet
ASM: Active shape model
ACM: Active contour mode
ATASM: Adaptive texture-based ASM
DSC: Dice similarity coefficient
CNN: Convolutional neural network
FCN: Fully convolutional network
FC-DenseNet: Fully convolutional DenseNet
GM: Gray matter
DenseASPP: Densely connected Atrous Spatial Pyramid Pooling
MB: Modeling-building
DFC-DN: Dilated FC-DenseNet
MAD: Mean of absolute distance
HD: Hausdorff distance
CHD: Convex hull Hausdorff distance

## References

[1] Makkouk AH, Oetgen ME, Swigart CR, Dodds SD (2018). Trigger finger: etiology, evaluation, and treatment. Curr Rev Musculoskelet Med..

[2] Doyle JR (1988). Anatomy of the finger flexor tendon sheath and pulley system. J Hand Surg..

[3] Ryzewicz M, Wolf JM (2006). Trigger digits: principles, management, and complications. J Hand Surg..

[4] Corley FG. Trigger Finger. Current Orthopedic diagnosis & treatment. 2000; 188-9.

[5] Sato J, Ishii Y, Noguchi H (2016). Comparison of the thickness of pulley and flexor tendon between in neutral and in flexed positions of trigger finger. Open Orthop J..

[6] Yang TH, Lin YH, Chuang BI, Chen HC, Lin WJ, Yang DS (2016). Identification of the position and thickness of the first annular pulley in sonographic images. Ultrasound Med Biol..

[7] Kim SJ, Lee CH, Choi WS, Lee BG, Kim JH, Lee KH (2016). The thickness of the A2 pulley and the flexor tendon are related to the severity of trigger finger: results of a prospective study using high-resolution ultrasonography. J Hand Surg..

[8] Manbachi A, Cobbold RS, Ginsberg HJ (2014). Guided pedicle screw insertion: techniques and training. Spine J..

[9] Pan JJ, Chang J, Yang X, Zhang JJ, Qureshi T, Howell R (2011). Graphic and haptic simulation system for virtual laparoscopic rectum surgery. Int J Med Robotics Comput Assist Surg..

[10] Zahiri M, Booton R, Nelson CA, Oleynikov D, Siu KC (2018). Virtual reality training system for anytime/anywhere acquisition of surgical skills: a pilot study. Milit Med.

[11] Zhu L, Ye X, Ji’er X, Gu Y, Guo S. A real-time deformation modeling scheme of soft tissue for virtual surgical. In: Proceedings of the 2010 IEEE international conference on information and automation, Harbin, China. 2010; p. 771-5. 10.1109/icinfa.2010.5512470.

[12] Gupta R, Elamvazuthi I, Dass SC, Faye I, Vasant P, George J (2014). Curvelet based automatic segmentation of supraspinatus tendon from ultrasound image: a focused assistive diagnostic method. Biomed Eng Online..

[13] Hamarneh G, Gustavsson T. Combining snakes and active shape models for segmenting the human left ventricle in echocardiographic images. In: Computers in cardiology, Cambridge, MA, USA. 2000; p. 115-8. 10.1109/cic.2000.898469.

[14] Cunningham RJ, Harding PJ, Loram ID (2017). Real-time ultrasound segmentation, analysis and visualisation of deep cervical muscle structure. IEEE Trans Med Imaging..

[15] Martins N, Sultan S, Veiga D, Ferreira M, Teixeira F, Coimbra M (2018). A new active contours approach for finger extensor tendon segmentation in ultrasound images using prior knowledge and phase symmetry. IEEE J Biomed Health inform..

[16] Chuang BI, Kuo LC, Yang TH, Su FC, Jou IM, Lin WJ (2017). A medical imaging analysis system for trigger finger using an adaptive texture-based active shape model (ATASM) in ultrasound images. PloS ONE..

[17] Long J, Shelhamer E, Darrell T. Fully convolutional networks for semantic segmentation. In: Proceedings of the IEEE conference on computer vision and pattern recognition. 2015; pp. 3431–3440. 10.1109/TPAMI.2016.2572683 27244717

[18] Badrinarayanan V, Kendall A, Cipolla R (2015). Segnet: a deep convolutional encoder-decoder architecture for image segmentation. IEEE Trans Pattern Anal Mach Intell..

[19] Ronneberger O, Fischer P, Brox T. U-net: Convolutional networks for biomedical image segmentation. In: International conference on medical image computing and computer-assisted intervention. 2015; pp. 234–241. 10.1007/978-3-319-24574-4_28.

[20] Huang G, Liu Z, Van Der Maaten L, Weinberger KQ. Densely Connected Convolutional Networks. In: CVPR. 2017; 1(2):4702:4708.

[21] Jégou S, Drozdzal M, Vazquez D, Romero A, Bengio Y. The one hundred layers tiramisu: fully convolutional densenets for semantic segmentation. In: Computer vision and pattern recognition Workshops (CVPRW). 2017; pp. 1175-1183.

[22] Kuok CP, Tsai BS, Yang TH, Su FC, Jou IM, et al. Automatic finger tendon segmentation from ultrasound images using deep learning. In: International computer symposium, Taiwan. 2018.

[23] Yu F, Koltun V. Multi-scale context aggregation by dilated convolutions. 2015; arXiv:1511.07122.

[24] Javaid U, Dasnoy D, Lee JA. Multi-organ segmentation of chest CT images in radiation oncology: comparison of standard and dilated UNet. In: International conference on advanced concepts for intelligent vision systems. 2018; p. 188-99. 10.1007/978-3-030-01449-0_16.

[25] Perone CS, Calabrese E, Cohen-Adad J (2018). Spinal cord gray matter segmentation using deep dilated convolutions. Sci Rep..

[26] Zhou B, Li Y, Wang J. A weakly supervised adaptive DenseNet for classifying thoracic diseases and identifying abnormalities. 2018. arXiv:1807.01257.

[27] Yang M, Yu K, Zhang C, Li Z, Yang K. DenseASPP for semantic segmentation in street scenes. In: Proceedings of the IEEE conference on computer vision and pattern recognition. 2018. p. 3684-92.

[28] Lee CY, Xie S, Gallagher P, Zhang Z, Tu Z. Deeply-supervised nets. In: Proceedings of the 18^th^ international conference on artificial intelligence and statistics, San Diego, CA, USA. 2015. p. 562-70.

[29] Mo J, Zhang L (2017). Multi-level deep supervised networks for retinal vessel segmentation. Int J CARS..

[30] Chung M, Lee J, Lee M, Lee J, Shin Y G. Deeply self-supervising edge-to-contour neural network applied to liver segmentation. 2018. arXiv:1808.00739.

[31] Lei Y, Wang T, Wang B, He X, Tian S, Jani AB, et al. Ultrasound prostate segmentation based on 3D V-Net with deep supervision. In: Medical imaging 2019: ultrasonic imaging and tomography, San Diego, CA, USA. 2019. 10.1117/12.2512558.

[32] Nurzynska K (2018). Deep learning as a tool for automatic segmentation of corneal endothelium images. Symmetry..

[33] Lorensen WE, Cline HE (1987). Marching cubes: a high resolution 3D surface construction algorithm. ACM SIGGRAPH computer graphics..

[34] Huang G, Liu S, van der Maaten L, Weinberger KQ. CondenseNet: an efficient DenseNet using learned group convolutions. In: Proceedings of the IEEE conference on computer vision and pattern recognition. 2017. p. 2752-61.

[35] He K, Zhang X, Ren S, Sun J. Delving deep into rectifiers: surpassing human-level performance on ImageNet classification. In: Proceedings of the IEEE international conference on computer vision. 2015; p. 1026-34.

[36] Chen LC, Papandreou G, Schroff F, Adam H. Rethinking atrous convolution for semantic image segmentation. 2017. arXiv:1706.05587.

[37] Wang B, Lei Y, Tian S, Wang T, Liu Y, Patel P (2019). Deeply supervised 3D fully convolutional networks with group dilated convolution for automatic MRI prostate segmentation. Med Phys..

[38] Milletari F, Navab N, Ahmadi S-A. V-net: fully convolutional neural networks for volumetric medical image segmentation. In: 2016 fourth international conference on 3D vision (3DV). 2016. p. 565-71. 10.1109/3dv.2016.79.

